# Efficacy and safety of HLX01 in patients with moderate-to-severe rheumatoid arthritis despite methotrexate therapy: a phase 3 study

**DOI:** 10.1186/s13075-022-02821-x

**Published:** 2022-06-10

**Authors:** Xiaofeng Zeng, Ju Liu, Xiumei Liu, Lijun Wu, Yi Liu, Xiangping Liao, Huaxiang Liu, Jiankang Hu, Xin Lu, Linjie Chen, Jian Xu, Zhenyu Jiang, Fu-ai Lu, Huaxiang Wu, Ying Li, Qingyu Wang, Jun Zhu, the HLX01-RA03 Investigators, Lingyun Sun, Meimei Wang, Xiaoxia Yu, Pingting Yang, Qinghua Zou, Baijie Xu, Hua Zhang, Cibo Huang, Liqi Bi, Xiaoxia Li, Jianzhao Cheng, Hua Wei, Lan He, Hao Zhang, Hongsheng Sun, Zongwen Shuai, Jianhong Zhao, Yang Li, Rongbin Li, Fengju Li, Xiaomei Li, Zhuoli Zhang, Wufang Qi, Hongwei Du, Jingchun Jin, Jian Wu

**Affiliations:** 1 Department of Rheumatology, Peking Union Medical College Hospital, Beijing, China; 2 Department of Rheumatology, Jiujiang No. 1 People’s Hospital, Jiujiang, China; 3 Department of Rheumatology, First Hospital of Shanxi Medical University, Taiyuan, China; 4 Department of Rheumatology, People’s Hospital of Xinjiang Uygur Autonomous Region, Urumqi, China; 5 Department of Rheumatology, West China Hospital of Sichuan University, Chengdu, China; 6 Department of Nephrology, Chenzhou First People’s Hospital, Chenzhou, China; 7 Department of Rheumatology, Qilu Hospital of Shandong University, Jinan, China; 8 Department of Rheumatology, Jiangxi Pingxiang People’s Hospital, Pingxiang, China; 9 Department of Rheumatology, China-Japan Friendship Hospital, Beijing, China; 10 Department of Rheumatology, The First Affiliated Hospital of Bengbu Medical College, Bengbu, China; 11 Department of Rheumatology and Immunology, First Affiliated Hospital of Kunming Medical University, Kunming, China; 12 Department of Rheumatology, The First Hospital of Jilin University, Changchun, China; 13 Department of Rheumatology, The First Affiliated Hospital of Baotou Medical College, Inner Mongolia University of Science and Technology, Baotou, China; 14 Department of Rheumatology, The Second Affiliated Hospital Zhejiang University School of Medicine, Hangzhou, China; 15 Shanghai Henlius Biotech, Inc., Shanghai, China

**Keywords:** Rheumatoid arthritis, Rituximab, Methotrexate, Phase 3, Biologic disease-modifying anti-rheumatic drug

## Abstract

**Background:**

To evaluate the efficacy and safety of HLX01, a rituximab biosimilar, as combination therapy with methotrexate in Chinese patients with active rheumatoid arthritis who had inadequate responses to methotrexate.

**Methods:**

In this double-blind, placebo-controlled phase 3 trial, biologic-naïve patients with moderate-to-severe active rheumatoid arthritis and inadequate responses to methotrexate were randomized 2:1 to receive 1000 mg HLX01 or placebo intravenously on days 1 and 15. On the first day of weeks 24 and 26, patients in both groups received 1000 mg HLX01 via intravenous infusion. The primary endpoint was the American College of Rheumatology (ACR) 20 response rate at week 24. Secondary endpoints including efficacy, safety, immunogenicity, pharmacokinetics and pharmacodynamics were assessed up to week 48.

**Results:**

Between 28 May 2018 and 11 September 2020, 275 patients were randomized to the HLX01 group (*n* = 183) or the placebo group (*n* = 92). At week 24, the proportion of patients achieving ACR20 response was significantly greater in the HLX01 group compared with the placebo group in the intention-to-treat population (60.7% *vs* 35.9%; *P* < 0.001) and per-protocol set (60.3% *vs* 37.1%; *P* < 0.001). Most secondary efficacy endpoints favoured HLX01 when assessed at weeks 12, 24, 36 and 48. Incidences of treatment-emergent adverse events were similar between groups. Infusion-related reactions occurred more frequently following the initial two doses of HLX01 than the subsequent doses.

**Conclusions:**

HLX01 plus methotrexate improved clinical outcomes compared with placebo in Chinese patients with rheumatoid arthritis who had inadequate responses to methotrexate. This treatment regimen was well tolerated, showing comparable safety profiles to placebo.

**Trial registration:**

ClinicalTrials.gov, NCT03522415. Registered on 11 May 2018.

**Supplementary Information:**

The online version contains supplementary material available at 10.1186/s13075-022-02821-x.

## Background

Rheumatoid arthritis (RA) is associated with persistent joint pain and stiffness that greatly impacts physical function and quality of life [1]. Clinical remission or, less desirably, low disease activity (LDA), is the principal treatment goal in patients with RA [2]. Currently, multiple treatment options are available for RA patients, including conventional synthetic (cs) disease-modifying anti-rheumatic drugs (DMARDs; e.g. methotrexate [MTX] and leflunomide), targeted synthetic DMARDs (mainly JAK inhibitors, such as tofacitinib and baricitinib), tumour necrosis factor (TNF)-inhibiting biologics (e.g. adalimumab and etanercept) and non-TNF-inhibiting biologics (e.g. abatacept and rituximab) [3]. MTX is a csDMARD recommended as a first-line treatment for RA [2, 4]. However, up to 40% of RA patients are refractory or respond insufficiently to MTX and thus require an alternative therapeutic strategy [5]. For these patients, biologic DMARDs in combination with csDMARDs are recommended by the current guidelines [2, 4, 6].

Rituximab is a chimeric antibody directed against the CD20 antigen on the surface of normal and malignant B lymphocytes [7]. Based on data from international registries, systematic reviews and meta-analyses, rheumatoid factor (RF) and/or anti-citrullinated protein antibody (ACPA, of which the presence and levels are frequently defined by anti-cyclic citrullinated peptide [anti-CCP] antibody) could be useful biomarkers for choosing B-cell depleting therapy with rituximab as a second-line treatment in RA patients who have failed the first biologic agent [8]. Rituximab is approved as a biologic DMARD in combination with MTX by the US Food and Drug Administration and the European Medicines Agency for the treatment of adult RA patients who show inadequate responses or intolerance to TNF inhibitors [7, 9]. The combination of rituximab and MTX has shown superior efficacy over placebo in patients refractory to TNF inhibitors [10–12] and in biologic-naïve patients responding poorly to MTX [13, 14]. In routine practice, rituximab requires less frequent dosing [15] and is associated with a lower rate of discontinuation than TNF inhibitors [16].

To date, rituximab has not been approved in China for RA treatment. HLX01 (Shanghai Henlius Biotech, Inc.) is a rituximab biosimilar that has demonstrated bioequivalence to reference rituximab in physical and biochemical properties [17], in pharmacokinetics (PK), pharmacodynamics (PD), safety and preliminary efficacy in RA patients in a phase 1/2 trial [18], and in efficacy and safety in patients with diffuse large B-cell lymphoma (DLBCL) in a phase 3 trial [19]. HLX01 has been approved in China as the first biosimilar for the same indications as the rituximab originator in non-Hodgkin’s lymphoma and chronic lymphocytic leukaemia [20, 21]. Approval of HLX01 in China for treating RA would expand therapeutic options for Chinese patients. Therefore, we conducted this phase 3 trial of HLX01 *vs* placebo in addition to background MTX to evaluate its efficacy and safety in Chinese patients with moderate-to-severe active RA responding poorly to prior MTX therapy.

## Methods

### Trial design and treatment

**Fig. 1:**
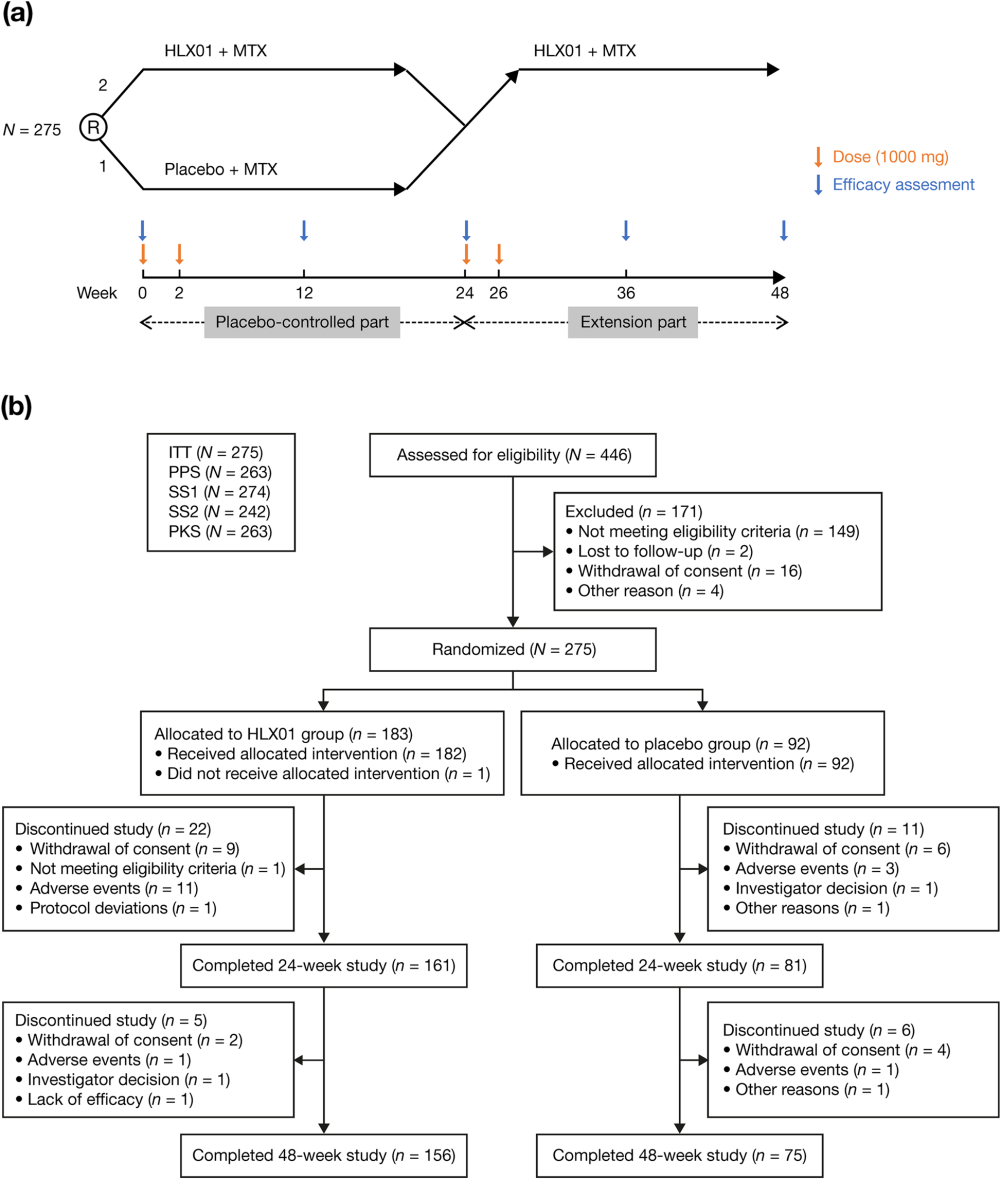
Study design and patient flow chart. **a** Study design. **b** Patient flow chart. ITT intention-to-treat, MTX methotrexate, PKS pharmacokinetic set, PPS per-protocol set, R randomized, SS1 safety set 1, SS2 safety set 2

This study was a randomized, double-blind, placebo-controlled phase 3 trial, conducted from 28 May 2018 to 11 September 2020 at 40 centres in China (Additional file 1, Table S1). The study consisted of two parts: a placebo-controlled part (day 1 to week 24) and an extension part (weeks 24 to 48) (Fig. 1a). In the placebo-controlled part, patients were randomly assigned (2:1) to receive either 1000 mg HLX01 or placebo via intravenous infusion on day 1 (first day of week 0) and day 15 (first day of week 2). During the extension part, 1000 mg HLX01 was administered on day 169 (first day of week 24) and day 183 (first day of week 26) to patients who initially received HLX01 or placebo. A stable dose of MTX (10–25 mg/week) was administered to all patients during the study (prespecified pre-medications, concomitant medications and rescue treatments are provided in Additional file 1, Data S1). Patients were randomized to groups by the investigators using an interactive web response system, with a randomization table generated by the statisticians. The investigators, patients and other study-related personnel remained blinded to the treatment allocation up to week 48.

This study was conducted in accordance with the principles of the Declaration of Helsinki, International Conference on Harmonisation Good Clinical Practice guidelines, and local applicable regulatory requirements [22–24]. The study protocol and informed consent were approved by the independent ethics committee at each participating site. All participants provided written informed consent. The trial was registered with ClinicalTrials.gov (NCT03522415).

### Patients

Eligible patients were aged 18–75 years, had an RA diagnosis for ≥6 months according to the American Rheumatism Association 1987 revised criteria [25] and had moderate-to-severe active disease (disease activity score of 28 joints [DAS28]-C-reactive protein [CRP] >3.2, ≥6 swollen joints based on a 66-joint count, and ≥6 tender joints based on a 68-joint count). Patients had to be on MTX (10–25 mg/week) treatment for ≥12 weeks and at a stable dose for ≥4 weeks, before treatment initiation (day 1). Patients who received other DMARDs, oral glucocorticoids, non-steroidal anti-inflammatory drugs (NSAIDs) or non-NSAID analgesics were eligible if the treatment met predefined conditions. Key exclusion criteria included prior treatment with biologic DMARDs; evidence or a history of tuberculosis; evidence or a history of inflammatory joint diseases other than RA or systemic autoimmune diseases; a history of malignancy (solid tumours, haematological malignancies, carcinoma in situ); and any active infections or a history of chronic, opportunistic or serious infections (see Additional file 1, Data S2 for detailed eligibility criteria).

### Study endpoints and assessments

The primary endpoint was the proportion of patients achieving an American College of Rheumatology (ACR) 20 response [26] at week 24. Secondary efficacy endpoints included the proportions of patients who achieved an ACR20/50/70 response assessed at weeks 12, 24, 36 and 48; change in disease activity assessed by DAS28-CRP and DAS28-erythrocyte sedimentation rate (ESR); the proportion of patients achieving clinical remission (defined as DAS28 ≤2.6) or LDA (DAS28 ≤3.2); improvement in arthritis pain, assessed by Patient’s Assessment of Arthritis Pain-Visual Analogue Scale (PtAAP-VAS); and improvement in physical function, assessed by Health Assessment Questionnaire-Disability Index (HAQ-DI).

Safety and tolerability were evaluated up to week 48. Clinical laboratory tests and physical assessments were performed, and vital signs were recorded at every visit. Treatment-emergent adverse events (TEAEs) were coded with the Medical Dictionary for Regulatory Activities (MedDRA) v23.0 and graded according to Common Terminology Criteria for Adverse Events v4.03. Predefined adverse events of special interest (AESIs) are listed in Additional file 1, Data S3.

Exploratory endpoints included change in physical and mental health summary scores (Short Form 36 Health Survey [SF-36] physical component summary [PCS] and mental component summary [MCS]), PK, immunogenicity and PD. Sampling time points for PK, immunogenicity and PD assessments are provided in Additional file 1, Data S4. Serum drug concentrations were measured by a validated enzyme-linked immunosorbent assay. Immunogenicity was assessed by antidrug antibodies (ADAs) and neutralizing antibodies (NAbs) against HLX01, measured using a Meso Scale Discovery electrochemiluminescent immunoassay. ADA or NAb positivity was defined as ≥1 positive result post-baseline. PD parameters included RF, CRP, ESR and anti-CCP antibodies. Based on laboratory tests, RF status was classified as positive (≥14 IU/ml) or negative, and anti-CCP status was classified as positive (>10 U/ml), negative (<7 U/ml) or unsure.

PK and immunogenicity parameters were analysed at a central laboratory (WuXi AppTec Co., Ltd., Shanghai, China). PD parameters were analysed at another central laboratory (Q2 Solutions Co., Ltd., Beijing, China) except for ESR, which was analysed at individual study centres.

### Statistical analysis

A sample size of 267 patients (randomized 2:1, i.e. 178 in the HLX01 group and 89 in the placebo group) was estimated to provide 80% power at a two-sided alpha level of 0.05 for the comparison of the ACR20 response rate between the two groups at week 24, assuming response rates of 50% in the HLX01 group and 30% in the placebo group, with a 20% dropout rate.

Primary endpoint and subgroup analyses stratified by RF and ADA status, secondary efficacy endpoints, PCS, MCS and PD were analysed on the intention-to-treat (ITT) population, which included all randomized patients. Sensitivity analyses on the efficacy endpoints were performed on the per-protocol set (PPS), which comprised all patients in the ITT population who received ≥1 dose of study medication and had no major protocol violations. The safety set 1 (SS1) and the safety set 2 (SS2) included all patients who received ≥1 dose of study medication in the placebo-controlled part (up to week 24) and in the extension part (weeks 24 to 48), respectively. PK analysis was based on the pharmacokinetic set (PKS), comprising all randomized patients who received ≥1 dose of HLX01 and provided ≥1 PK sample analysed as planned, without major protocol deviations that might impact PK assessment. Immunogenicity was analysed for patients within the SS1 who had ≥1 ADA or NAb assessment after receiving study medication.

If a patient received rescue treatment, all efficacy data collected from this patient thereafter were considered as missing data in statistical analyses. Comparisons of categorical efficacy measures between groups were performed using logistic regression, where missing data were handled using nonresponse imputation. Comparisons of continuous efficacy measures were conducted using analysis of covariance (ANCOVA) with adjustment for baseline assessments, in which the last-observation-carried-forward method was used to impute missing data. Data for other endpoints were summarized by descriptive statistics. All statistical analyses were performed using SAS^®^ software, v9.3 (SAS Institute Inc., Cary, NC, USA).

## Results

### Patients

Of the 446 patients screened, 275 were randomly assigned to the HLX01 group (*n* = 183) or the placebo group (*n* = 92) and comprised the ITT population. The most common reason for screen failure was not meeting eligibility criteria (*n* = 149). The PPS included 263 patients (HLX01, *n* = 174; placebo, *n* = 89). Reasons for exclusion from the PPS were major protocol violations (*n* = 11) and not receiving study medication (*n* = 1). One patient not receiving treatment on days 1 and 15 was excluded from the SS1 (HLX01, *n* = 182; placebo, *n* = 92), and patients not treated at weeks 24 and 26 were excluded from the SS2 (HLX01, *n* = 161; placebo, *n* = 81). The PKS included 263 patients (HLX01, *n* = 182; placebo, *n* = 81), and the reason for exclusion was not receiving HLX01 treatment.

In the ITT population, 274 (99.6%) patients completed treatment on day 1 (HLX01, *n* = 182; placebo, *n* = 92) and 265 (96.4%) patients completed treatment on day 15 (HLX01, *n* = 175; placebo, *n*=90). Two hundred forty-two (88.0%) patients completed treatment on week 24, with 161 from the HLX01 group and 81 from the placebo group. One hundred fifty-eight and 75 patients (233 in total [84.7%]) from the HLX01 group and the placebo group, respectively, completed treatment on week 26, and 156 and 75 patients completed the study on week 48 (Fig. 1b).

Baseline characteristics were well balanced between the HLX01 and placebo groups (Table 1). Most patients were female (84.7%) and RF positive (92.0%), with a mean DAS28-CRP of 5.5. The mean duration of disease was 88.4 *vs* 75.5 months in the HLX01 and placebo groups, respectively.

**Table 1:** Demographics and clinical characteristics at baseline

	HLX01 (***n*** = 183)	Placebo (***n*** = 92)
Age, years, mean (SD)	49.1 (11.8)	47.9 (11.0)
Female, *n* (%)	153 (83.6)	80 (87.0)
Chinese, *n* (%)	183 (100)	92 (100)
BMI, kg/m^2^, mean (SD)	22.7 (3.0)	22.2 (3.2)
Duration of disease, months, mean (SD)	88.4 (87.7)	75.5 (91.5)
RF positive, *n* (%)	167 (91.3)	86 (93.5)
Anti-CCP antibody positive, *n* (%)	175 (95.6)	81 (88.0)
Anti-CCP antibody negative, *n* (%)	7 (3.8)	9 (9.8)
SJC, mean (SD)	11.8 (5.9)	11.4 (7.3)
TJC, mean (SD)	20.9 (13.1)	19.5 (12.7)
CRP, mg/l, mean (SD)	16.9 (21.0)	22.2 (27.6)
ESR, mm/h, mean (SD)	39.2 (24.6)	43.4 (28.1)
DAS28-CRP, mean (SD)	5.5 (0.9)	5.4 (1.0)
DAS28-ESR, mean (SD)	6.1 (1.1)	6.1 (1.1)
HAQ-DI score, mean (SD)	1.4 (0.7)	1.5 (0.7)
PtAAP-VAS score, mean (SD)	60.5 (21.3)	58.2 (21.5)

*Anti-CCP* anti-cyclic citrullinated peptide, *BMI* body mass index, *CRP* C-reactive protein, *DAS28* disease activity score of 28 joints, *ESR* erythrocyte sedimentation rate, *HAQ-DI* Health Assessment Questionnaire-Disability Index, *PtAAP-VAS* Patient’s Assessment of Arthritis Pain-Visual Analogue Scale, *RF* rheumatoid factor, *SD* standard deviation, *SJC* swollen joint count, *TJC* tender joint count

### Efficacy

**Fig. 2:**
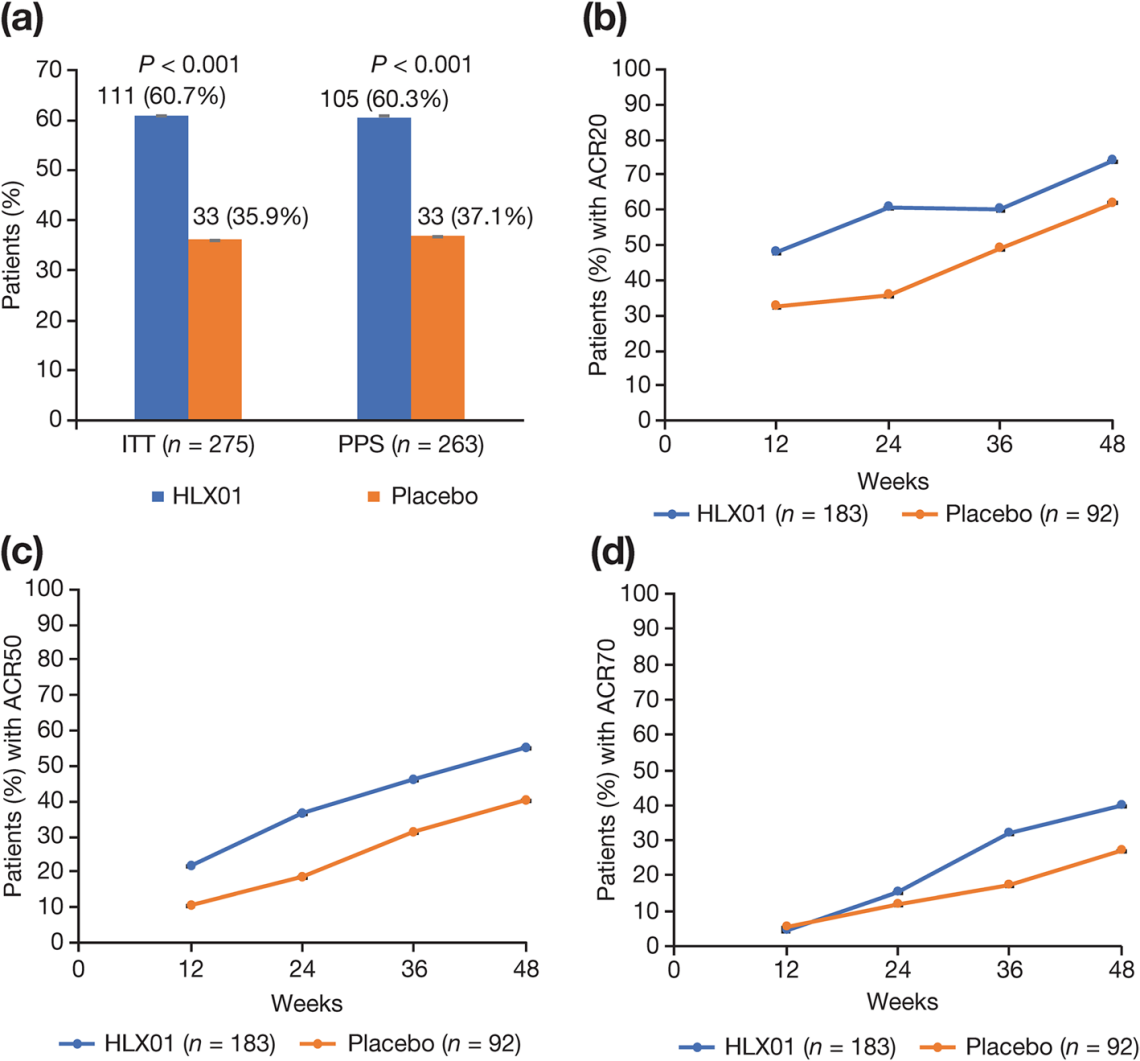
ACR20, ACR50 and ACR70 responses. **a** Proportion of patients with an ACR20 response in the ITT population and the PPS population at week 24, and proportion of patients achieving a clinical response according to **b** ACR20, **c** ACR50 and **d** ACR70 in the ITT population over time. Error bars represent standard error. ACR American College of Rheumatology, ITT intention-to-treat, PPS per-protocol set

A significantly greater proportion of patients achieved ACR20 response in the HLX01 group compared with the placebo group in the ITT population at week 24 (60.7% *vs* 35.9%), with an odds ratio (OR) of 2.8 (95% confidence interval [CI] 1.6, 4.6) and a *P* value of <0.001 (Fig. 2a). The ACR20 response rate was also significantly greater in the HLX01 group than in the placebo group in the PPS (60.3% *vs* 37.1%; OR 2.6 [95% CI 1.5, 4.4]; *P* < 0.001). Subgroup analyses by RF and ADA status showed a greater ACR20 response rate in the HLX01 group compared with the placebo group among RF-positive patients and among ADA-negative patients, whereas between-group differences were inconclusive in RF-negative and in ADA-positive patients due to the small sample size (Additional file 1, Table S2).

A greater ACR20 response rate in the HLX01 group compared with the placebo group was observed in the ITT population at week 12 (48.1% *vs* 32.6%; OR 1.9 [95% CI 1.1, 3.2]; Fig. 2b). After week 24, the ACR20 response rate was at similar or higher levels than that at week 24 in patients who continued HLX01 treatment. In patients who switched to HLX01 from placebo at week 24, the response rate progressively increased. The ACR50 response rate was greater in the HLX01 group compared with the placebo group at week 12 (21.9% *vs* 10.9%; OR 2.3 [95% CI 1.1, 4.8]) and week 24 (36.6% *vs* 18.5%; OR 2.5 [95% CI 1.4, 4.7]), which increased further after week 24 in both groups. ACR70 was similar between the two groups at weeks 12 and 24 and increased after week 24 in both groups (Fig. 2c, d).

**Fig. 3:**
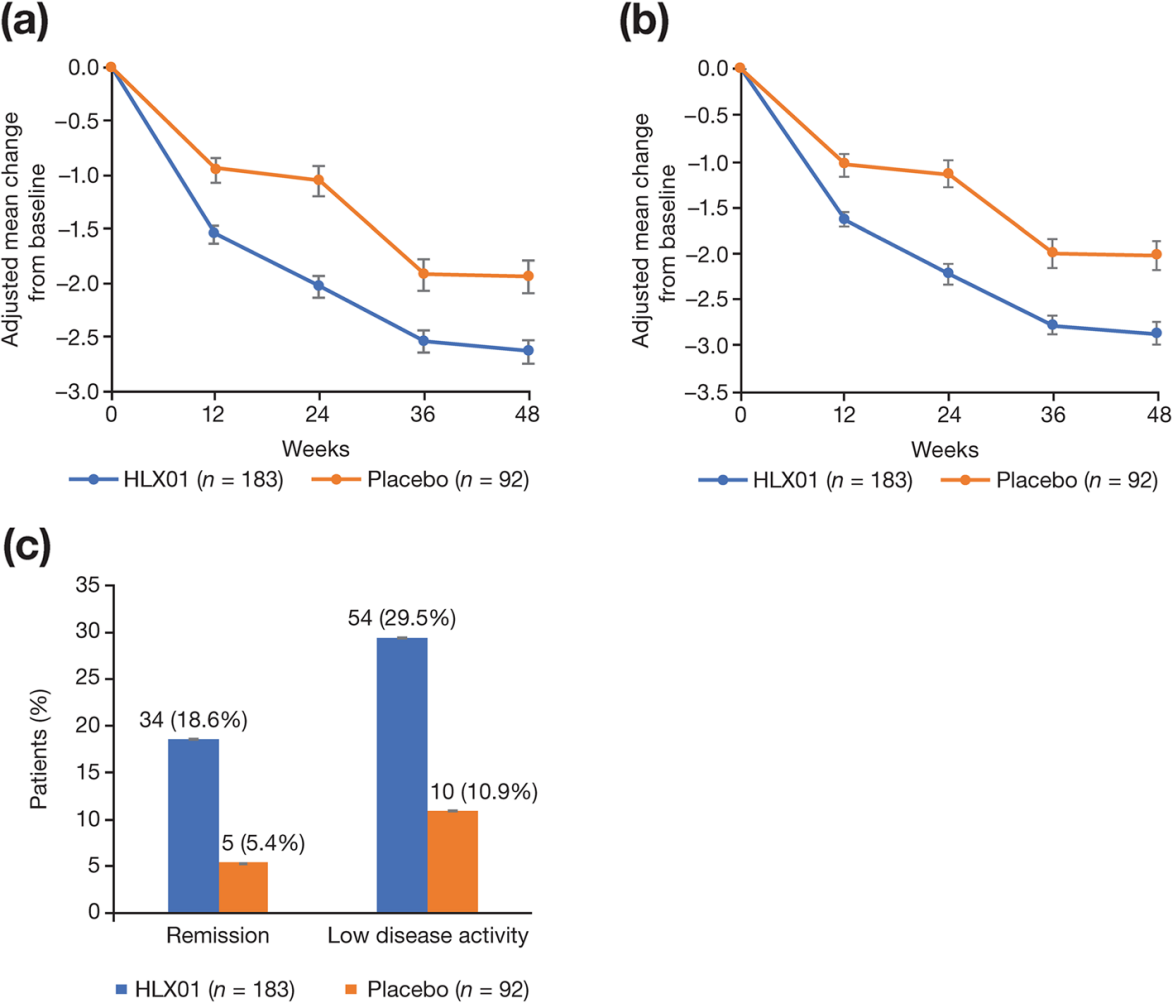
Change in disease activity based on DAS28-CRP and DAS28-ESR. **a** DAS28-CRP and **b** DAS28-ESR adjusted mean change from baseline in the ITT population. **c** Proportion of patients with remission (DAS28 ≤2.6) or low disease activity (DAS28 ≤3.2) at week 24 in the ITT population. Error bars represent standard error. CRP C-reactive protein, DAS28 disease activity score of 28 joints, ESR erythrocyte sedimentation rate, ITT intention-to-treat

Patients treated with HLX01 showed greater reductions in DAS28-CRP (adjusted mean change: −2.0 *vs* −1.1) and DAS28-ESR (adjusted mean change: −2.2 *vs* −1.1) compared with placebo in the ITT population at week 24 (Fig. 3a, b). After week 24, these disease activity measures continued to decrease in both groups during HLX01 treatment. Greater proportions of patients in the HLX01 group than the placebo group achieved remission (DAS28 ≤ 2.6: 18.6% *vs* 5.4%; OR 4.0 [95% CI 1.5, 10.5]) or LDA (DAS28 ≤3.2, 29.5% *vs* 10.9%; OR 3.4 [95% CI 1.7, 7.1]) at week 24 (Fig. 3c), and both proportions in each group increased further up to week 48 (Additional file 1, Fig. S1).

Arthritis pain (PtAAP-VAS), physical function (HAQ-DI) and physical and mental health (SF-36 PCS and MCS) improved to a greater extent in the HLX01 group than in the placebo group in the ITT population up to week 24, and all showed a continuous improvement thereafter in the two groups (Additional file 1, Fig. S2).

Analyses of efficacy endpoints in the PPS showed similar results to those in the ITT population.

### Pharmacokinetics

In patients who received HLX01 before week 24 and continued HLX01 treatment after week 24, post-dose HLX01 serum concentration increased by 27% on day 15 *vs* day 1 and by 23% on day 183 *vs* day 169, indicating an accumulation of HLX01 in serum. HLX01 was detectable in the serum at week 12 (day 85) and week 36 (day 253), at a concentration ≤5% of that immediately post-dose on days 15 and 183. After week 24, the dynamics of serum HLX01 in patients who switched to HLX01 from placebo were similar to those who continued HLX01 treatment. Serum drug concentrations and trends were similar between the two treatment courses (Additional file 1, Fig. S3).

### Immunogenicity

In general, the percentage of patients with ≥1 positive post-baseline ADA result was very low. ADAs were detected in 11 (6.0%) and 3 (3.3%) patients in the HLX01 group and the placebo group before week 24, respectively, and in 7 (3.8%) and 8 (8.8%) patients who continued HLX01 or switched to HLX01 after week 24. Only one patient who switched to HLX01 tested positive for NAb (i.e. during weeks 24–48 [day 182]). In ADA-positive patients *vs* ADA-negative patients, the peak drug concentrations were similar, while the trough drug concentration and drug concentration at weeks 12 and 36 were lower in ADA-positive patients (Additional file 1, Fig. S4).

### Pharmacodynamics

Among patients seropositive for RF at baseline, 3 (1.7%) and 1 (1.1%) in the HLX01 and placebo groups, respectively, tested negative for RF at all post-baseline assessments before week 24; 16 (8.9%) and 2 (2.2%) in the two treatment groups tested negative for RF during weeks 24–48. At week 24, 8 (4.9%) patients in the HLX01 group and 4 (5.1%) in the placebo group who were seropositive for anti-CCP antibodies at baseline became non-positive (i.e. negative and unsure). CRP and ESR were reduced more substantially in the HLX01 group than in the placebo group before week 24, and both were reduced further after week 24 in the two groups (Additional file 1, Fig. S5).

### Safety

Up to week 24, 151 (83.0%) patients in the HLX01 group and 74 (80.4%) in the placebo group reported ≥1 TEAE (Table 2). Most TEAEs in both groups were grade 1/2 in severity. The rates of serious TEAEs were similar between groups (6.6% *vs* 7.6%). TEAEs leading to treatment discontinuation (7.1% *vs* 4.3%) and AESIs (5.5% *vs* 2.2%) were more frequent in the HLX01 group than in the placebo group (Table 2; Additional file 1, Table S3). Infusion-related reactions (IRRs) occurred at a greater frequency in the HLX01 group than in the placebo group (12.1% *vs* 2.2%), but only one event (in the HLX01 group) was grade ≥3. The incidence of serious infections was comparable in the two groups (2.2% *vs* 2.2%). Among the AESIs, the most common events were immediate hypersensitivity reactions (1.6% *vs* 0) and infective pneumonia (1.1% *vs* 0); there was one (0.5%) diagnosis of pulmonary sclerosing pneumocytoma, a benign tumour, in the HLX01 group.

**Table 2:** Treatment-emergent adverse events

	Placebo-controlled part Up to week 24 (SS1)		Extension part Week 24 to week 48 (SS2)	
	HLX01 (***n*** = 182)	Placebo (***n*** = 92)	Continue HLX01 (***n*** = 161)	Switch to HLX01 from placebo (***n*** = 81)
Any TEAEs	151 (83.0)	74 (80.4)	129 (80.1)	68 (84.0)
Grade 1 or 2	132 (72.5)	66 (71.7)	115 (71.4)	59 (72.8)
Grade 3	17 (9.3)	8 (8.7)	12 (7.5)	9 (11.1)
Grade 4	2 (1.1)	0	2 (1.2)	0
Serious TEAEs	12 (6.6)	7 (7.6)	9 (5.6)	4 (4.9)
TEAEs leading to treatment discontinuation	13 (7.1)	4 (4.3)	1 (0.6)	3 (3.7)
ADRs	100 (54.9)	47 (51.1)	86 (53.4)	47 (58.0)
IRRs	22 (12.1)	2 (2.2)	8 (5.0)	6 (7.4)
Infections and infestations	52 (28.6)	24 (26.1)	35 (21.7)	11 (13.6)
AESIs	10 (5.5)	2 (2.2)	7 (4.3)	5 (6.2)
IRRs (grade ≥3)	1 (0.5)	0	0	0
Infections (grade ≥3)	4 (2.2)	2 (2.2)	1 (0.6)	2 (2.5)
Most common TEAEs^a^				
Upper respiratory tract infection	33 (18.1)	17 (18.5)	21 (13.0)	10 (12.3)
Urinary tract infection	20 (11.0)	8 (8.7)	16 (9.9)	2 (2.5)
Anaemia	13 (7.1)	8 (8.7)	6 (3.7)	6 (7.4)
Hypertriglyceridemia	12 (6.6)	6 (6.5)	13 (8.1)	11 (13.6)
Liver dysfunction	12 (6.6)	6 (6.5)	13 (8.1)	3 (3.7)
Leukopenia	10 (5.5)	0	14 (8.7)	6 (7.4)
Hypokalaemia	10 (5.5)	5 (5.4)	8 (5.0)	8 (9.9)
Lymphocyte count decreased	14 (7.7)	2 (2.2)	14 (8.7)	5 (6.2)

Data are presented as number (%) of patients

*ADR* adverse drug reaction, *AESI* adverse event of special interest, *IRR* infusion-related reaction, *SS1* safety set 1, *SS2* safety set 2, *TEAE* treatment-emergent adverse event

^a^Occurring in >8% of patients in either group

From week 24 to week 48, the incidences, types and severities of TEAEs in the two groups were broadly consistent with those reported in the HLX01 group before week 24 (Table 2). Notably, fewer patients experienced IRRs following the second course of HLX01 (first *vs* second course, 12.1% *vs* 5.0%), and none of these was grade ≥3. Four patients discontinued treatment because of TEAEs, including one (0.6%) who continued HLX01 and three (3.7%) who switched to HLX01. Serious infections occurred in one (0.6%) and two (2.5%) patients in these respective groups. Among the AESIs, infective pneumonia occurred in one (0.6%) patient who continued HLX01 and in one (1.2%; fungal pneumonia) who switched to HLX01; there were one (0.6%) case of active tuberculosis and one (0.6%) case of latent tuberculosis in patients who continued HLX01 and two (2.5%) cases of active tuberculosis among patients who switched to HLX01 (Additional file 1, Table S3). There were no deaths in either group throughout the study.

## Discussion

This is the first phase 3 trial of HLX01 as an add-on to MTX in patients with moderate-to-severe RA. We found that two infusions of HLX01 conferred a superior response compared with placebo in terms of ACR20 and other standard disease activity measures at week 24. This response was sustained by a second course of HLX01 treatment, following which improvements in disease activity persisted over 48 weeks. The incidences of TEAEs associated with HLX01 were comparable to placebo. This study provides evidence in support of the use of HLX01 as an add-on to MTX in biologic-naïve Chinese patients with moderate-to-severe active RA despite MTX treatment.

This study was not designed as a bioequivalence study, because of the lack of an approved anti-CD20 treatment for RA in China and the limited evidence available for rituximab in Chinese patients with RA. The similarity between HLX01 and reference rituximab has been established in preclinical investigations and in studies in RA and other indications [17–19]. The current trial provides further evidence that the clinical benefit of HLX01 is at least comparable to the originator rituximab. Although a direct comparison of HLX01 and TNF inhibitors was not included in this study, the ACR20 response rate at week 24 in patients treated with HLX01 was comparable to those treated with TNF inhibitors (ranging from 47.8 to 75.7%) in previously reported clinical studies [27, 28].

Both ACR20 and ACR50 favoured HLX01 at weeks 12 and 24, whereas ACR70 did not show significant differences between the HLX01 and placebo groups at these two time points. ACR70 is a less sensitive response criterion and thus may sometimes exclude clinically meaningful improvements in RA status [29]. In addition to the superior efficacy of HLX01 over placebo at week 24, we also showed that the clinical benefits became evident as early as week 12 and that after a second course of HLX01 treatment starting at week 24, improvements in disease activity were maintained or enhanced at weeks 36 and 48. This supports a dosing interval of every 24 weeks with HLX01, similar to that of rituximab.

Potential safety concerns associated with rituximab in the treatment of RA include IRRs, serious infections, malignancy and hypogammaglobulinemia [30]. The pattern and severity of IRRs associated with HLX01 administration were similar to those observed with rituximab [14] and were less frequent following the second course of treatment. All but one IRR in our study were grade 1/2, similar to the experience for patients with DLBCL in a previous phase 3 bioequivalence study (all grade 1/2) [19]. Serious infections occurred in ≤5% of patients in the HLX01 and placebo groups. Notably, there is a high risk of background infection among RA patients due to immune dysfunction and the use of immunosuppressant medications [31]. RA patients may be susceptible to tuberculosis infection in China due to the high incidence of tuberculosis [32]. In our study, one (0.4%) patient reported latent tuberculosis, and three (1.2%) reported active tuberculosis when treated with HLX01 in the extension treatment period. Rituximab is associated with a lower risk of tuberculosis than TNF inhibitors in RA patients (12 cases/100,000 patient-years *vs* 65 cases/100,000 patient-years, respectively) [33]. Whether HLX01 has a safety advantage over TNF inhibitors in terms of the likelihood of tuberculosis needs confirmation with long-term monitoring, as tuberculosis could be an important treatment consideration, especially in areas with high incidences. In this study, there were no cases of new malignancy or hypogammaglobulinemia-related TEAEs. The safety profile of HLX01 in patients with RA appears acceptable and typical of that associated with CD20 inhibition.

Although more evidences are needed for the best application of precision medicine principles, ACPA (or anti-CCP antibody) and RF are currently the key biomarkers in the management of RA. In our study, 92.0% and 93.1% of all randomized patients were seropositive for RF and anti-CCP antibodies at baseline, respectively. The proportion of patients turning seronegative for RF after treatment was slightly higher in the HLX01 group (HLX01 *vs* placebo: 1.7% *vs* 1.1% before week 24, 8.9% *vs* 2.2% after week 24), while for anti-CCP antibody the proportion of patients turning non-positive was similar between the two groups (HLX01 *vs* placebo, 4.9% *vs* 5.1%). This might be an indication of lower disease activity and better prognosis resulted from the addition of HLX01 to MTX in RA treatment, but the proportions were rather small to reach a conclusion.

This study has several limitations. Only Chinese patients were included, and thus, future studies with other ethnicities are recommended. Only 22 patients were RF negative, limiting the power of the subgroup analysis by RF. Also, a subgroup analysis of efficacy by anti-CCP antibody status, which has been shown to influence the clinical outcome of rituximab [14, 34], was not performed. However, the proportions of RA patients seronegative for RF and anti-CCP are low, probably due to the nature of the disease, and thus, expanding the sample size in future studies might be the best choice to provide enough number of patients for efficacy analysis stratified by RF and anti-CCP antibody status. Additionally, for the development of precision medicine in RA treatment, future studies could be designed to include efficacy analysis by novel biomarkers for disease activity and prognosis apart from RF and anti-CCP antibody.

## Conclusions

In conclusion, HLX01, given in two treatment courses, provides sustainable disease activity improvements over 48 weeks and has an acceptable safety profile in biologic-naïve patients who have moderate-to-severe active RA despite MTX treatment. This supports the use of HLX01 in Chinese patients with moderate-to-severe active RA after failure of MTX.

## Supplementary Information

**Additional file 1: Supplementary Data S1.** Pre-medications, concomitant medications and rescue treatments. **Supplementary Data S2.** Inclusion and exclusion criteria. **Supplementary Data S3.** Adverse events of special interest. **Supplementary Data S4.** Sampling timepoints for assessments of pharmacokinetics, immunogenicity and pharmacodynamics. **Supplementary Table S1.** The HLX01-RA03 Investigators and study centres. **Supplementary Table S2.** Subgroup analyses of ACR20 response rate at week 24 in the intention-to-treat population. **Supplementary Table S3.** Adverse events of special interest. **Supplementary Fig. S1.** Proportion of patients with remission or low disease activity at weeks 36 and 48. Proportion of patients with remission (DAS28 ≤2.6) or low disease activity (DAS28 ≤3.2) at (a) week 36 and (b) week 48 in the intention-to-treat population. Error bars represent standard error. DAS28: disease activity score of 28 joints. **Supplementary Fig. S2.** Change from baseline in patient-reported outcomes. Adjusted mean change from baseline in (a) PtAAP-VAS, (b) HAQ-DI scores, (c) SF-36 PCS, and (d) SF-36 MCS in the intention-to-treat population. Error bars represent standard error. HAQ-DI: Health Assessment Questionnaire-Disability Index; PtAAP-VAS: Patient’s Assessment of Arthritis Pain-Visual Analogue Scale; SF-36 MCS: Short Form 36 Health Survey mental component summary; SF-36 PCS: Short Form 36 Health Survey physical component summary. **Supplementary Fig. S3.** Serum concentrations of HLX01 over time. Mean serum concentration of HLX01 over time in the pharmacokinetic set, plotted on (a) a linear scale or (b) a semi-log scale. Error bars represent standard deviation. **Supplementary Fig. S4.** Serum concentrations of HLX01 over time stratified by antidrug antibody status. Mean serum concentration of HLX01 over time stratified by antidrug antibody status in the HLX01 group ([a] linear scale; [b] semi-log scale) or in the placebo group ([c] linear scale; [d] semi-log scale) in the pharmacokinetic set. Error bars represent standard deviation. **Supplementary Fig. S5.** Change from baseline in CRP and ESR over time. Adjusted mean change from baseline in (a) CRP and (b) ESR in the intention-to-treat population. Error bars represent standard error. CRP: C-reactive protein; ESR: erythrocyte sedimentation rate.

## Data Availability

The datasets used and/or analysed during the current study are available from the corresponding author upon reasonable request.

## Abbreviations

ACPA: Anti-citrullinated protein antibody
ACR: American College of Rheumatology
ADA: Antidrug antibody
AESI: Adverse event of special interest
ANCOVA: Analysis of covariance
Anti-CCP: Anti-cyclic citrullinated peptide
CI: Confidence interval
CRP: C-reactive protein
CS: Conventional synthetic
DAS28: Disease activity score of 28 joints
DLBCL: Diffuse large B-cell lymphoma
DMARD: Disease-modifying anti-rheumatic drug
ESR: Erythrocyte sedimentation rate
HAQ-DI: Health Assessment Questionnaire-Disability Index
IRR: Infusion-related reaction
ITT: Intention-to-treat
LDA: Low disease activity
MCS: Mental component summary
MedDRA: Medical Dictionary for Regulatory Activities
MTX: Methotrexate
NAb: Neutralizing antibody
NSAID: Non-steroidal anti-inflammatory drug
OR: Odds ratio
PCS: Physical component summary
PD: Pharmacodynamics
PK: Pharmacokinetics
PKS: Pharmacokinetic set
PPS: Per-protocol set
PtAAP-VAS: Patient’s Assessment of Arthritis Pain-Visual Analogue Scale
RA: Rheumatoid arthritis
RF: Rheumatoid factor
SF-36: Short Form 36 Health Survey
SS1: Safety set 1
SS2: Safety set 2
TEAE: Treatment-emergent adverse event
TNF: Tumour necrosis factor

## References

[1] McInnes IB, Schett G (2011). The pathogenesis of rheumatoid arthritis. N Engl J Med.

[2] Smolen JS, Landewe RBM, Bijlsma JWJ, Burmester GR, Dougados M, Kerschbaumer A (2020). EULAR recommendations for the management of rheumatoid arthritis with synthetic and biological disease-modifying antirheumatic drugs: 2019 update. Ann Rheum Dis.

[3] Mease PJ, Stryker S, Liu M, Salim B, Rebello S, Gharaibeh M (2021). Treatment patterns in rheumatoid arthritis patients newly initiated on biologic and conventional synthetic disease-modifying antirheumatic drug therapy and enrolled in a North American clinical registry. Arthritis Res Ther.

[4] Singh JA, Saag KG, Bridges SL, Akl EA, Bannuru RR, Sullivan MC (2016). 2015 American College of Rheumatology guideline for the treatment of rheumatoid arthritis. Arthritis Care Res (Hoboken).

[5] Aletaha D, Stamm T, Kapral T, Eberl G, Grisar J, Machold KP (2003). Survival and effectiveness of leflunomide compared with methotrexate and sulfasalazine in rheumatoid arthritis: a matched observational study. Ann Rheum Dis.

[6] Chinese Rheumatology Association (2018). 2018 Chinese guideline for the diagnosis and treatment of rheumatoid arthritis. Zhonghua Nei Ke Za Zhi.

[7] RITUXAN® prescribing information [Internet]. U.S. Food and Drug Administration [cited 8 February 2022]. Available from: https://www.accessdata.fda.gov/drugsatfda_docs/label/2021/103705s5467lbl.pdf.

[8] Giacomelli R, Afeltra A, Alunno A, Baldini C, Bartoloni-Bocci E, Berardicurti O (2017). International consensus: what else can we do to improve diagnosis and therapeutic strategies in patients affected by autoimmune rheumatic diseases (rheumatoid arthritis, spondyloarthritides, systemic sclerosis, systemic lupus erythematosus, antiphospholipid syndrome and Sjogren’s syndrome)?: the unmet needs and the clinical grey zone in autoimmune disease management. Autoimmun Rev.

[9] MabThera product information [Internet]. European Medicines Agency [cited 17 March 2021]. Available from: https://www.ema.europa.eu/en/medicines/human/EPAR/mabthera#authorisation-details-section.

[10] Cohen SB, Emery P, Greenwald MW, Dougados M, Furie RA, Genovese MC (2006). Rituximab for rheumatoid arthritis refractory to anti-tumor necrosis factor therapy: results of a multicenter, randomized, double-blind, placebo-controlled, phase III trial evaluating primary efficacy and safety at twenty-four weeks. Arthritis Rheum.

[11] Mease PJ, Cohen S, Gaylis NB, Chubick A, Kaell AT, Greenwald M (2010). Efficacy and safety of retreatment in patients with rheumatoid arthritis with previous inadequate response to tumor necrosis factor inhibitors: results from the SUNRISE trial. J Rheumatol.

[12] Cohen SB, Keystone E, Genovese MC, Emery P, Peterfy C, Tak PP (2010). Continued inhibition of structural damage over 2 years in patients with rheumatoid arthritis treated with rituximab in combination with methotrexate. Ann Rheum Dis.

[13] Edwards JC, Szczepanski L, Szechinski J, Filipowicz-Sosnowska A, Emery P, Close DR (2004). Efficacy of B-cell-targeted therapy with rituximab in patients with rheumatoid arthritis. N Engl J Med.

[14] Emery P, Deodhar A, Rigby WF, Isaacs JD, Combe B, Racewicz AJ (2010). Efficacy and safety of different doses and retreatment of rituximab: a randomised, placebo-controlled trial in patients who are biological naive with active rheumatoid arthritis and an inadequate response to methotrexate (Study Evaluating Rituximab’s Efficacy in MTX iNadequate rEsponders (SERENE)). Ann Rheum Dis.

[15] Wu B, Wilson A, Wang FF, Wang SL, Wallace DJ, Weisman MH (2012). Cost effectiveness of different treatment strategies in the treatment of patients with moderate to severe rheumatoid arthritis in China. PLoS One.

[16] Frisell T, Dehlin M, Di Giuseppe D, Feltelius N, Turesson C, Askling J et al. Comparative effectiveness of abatacept, rituximab, tocilizumab and TNFi biologics in RA: results from the nationwide Swedish register. Rheumatology (Oxford). 2019;58(8):1367–77. 10.1093/rheumatology/key433 30668875

[17] Xu Y, Xie L, Zhang E, Gao W, Wang L, Cao Y (2019). Physicochemical and functional assessments demonstrating analytical similarity between rituximab biosimilar HLX01 and the MabThera®. MAbs.

[18] Zeng X, Wang Y, Jiang Z, Zhang Z, He L, Zhang X et al. SAT0139 A multicentre, randomised, double-blind, parallel active-controlled clinical trial comparing pharmacokinetics, pharmacodynamics, safety and exploratory efficacy between HLX01 and Europe-sourced rituximab as a new indication in Chinese moderate to severe patients with rheumatoid arthritis. Ann Rheum Dis. 2019;78(Suppl 2):1140.

[19] Shi Y, Song Y, Qin Y, Zhang Q, Han X, Hong X (2020). A phase 3 study of rituximab biosimilar HLX01 in patients with diffuse large B-cell lymphoma. J Hematol Oncol.

[20] Rituximab injection (HLX01) product insert [Internet]. National Medical Products Administration [cited 02 July 2021]. Available from: https://www.henlius.com/upload/202008/13/%E6%B1%89%E5%88%A9%E5%BA%B7%C2%AE%EF%BC%88%E5%88%A9%E5%A6%A5%E6%98%94%E5%8D%95%E6%8A%97%E6%B3%A8%E5%B0%84%E6%B6%B2%EF%BC%89%E8%AF%B4%E6%98%8E%E4%B9%A6.pdf.

[21] Henlius receives NMPA approval for its first product HLX01 –the beginning of a new era for biosimilar [Internet] [cited 10 June 2021]. Available from: https://www.henlius.com/en/NewsDetails-1867-26.html.

[22] Statistical principles for clinical trails of chemical and biological agents [Internet]. National Medical Products Administration [cited 25 January 2021]. Available from: https://www.nmpa.gov.cn/wwwroot/gsz05106/10.pdf.

[23] Guidelines on Development and Evaluation of Biosimilars (drafting) [Internet]. National Medical Products Administration [cited 28 February 2021]. Available from: https://www.nmpa.gov.cn/yaopin/ypggtg/ypqtgg/20150228155701114.html.

[24] Provisions for drug registration [Internet]. National Medical Products Administration [cited 25 January 2021]. Available from: https://www.nmpa.gov.cn/xxgk/fgwj/bmgzh/20070710010101795.html.

[25] Arnett FC, Edworthy SM, Bloch DA, McShane DJ, Fries JF, Cooper NS (1988). The American Rheumatism Association 1987 revised criteria for the classification of rheumatoid arthritis. Arthritis Rheum.

[26] Felson DT, Anderson JJ, Boers M, Bombardier C, Furst D, Goldsmith C (1995). American College of Rheumatology. Preliminary definition of improvement in rheumatoid arthritis. Arthritis Rheum.

[27] Weinblatt ME, Keystone EC, Furst DE, Moreland LW, Weisman MH, Birbara CA (2003). Adalimumab, a fully human anti-tumor necrosis factor alpha monoclonal antibody, for the treatment of rheumatoid arthritis in patients taking concomitant methotrexate: the ARMADA trial. Arthritis Rheum.

[28] Genovese MC, Glover J, Greenwald M, Porawska W, El Khouri EC, Dokoupilova E (2019). FKB327, an adalimumab biosimilar, versus the reference product: results of a randomized, Phase III, double-blind study, and its open-label extension. Arthritis Res Ther.

[29] Ward MM, Guthrie LC, Alba MI (2014). Brief report: rheumatoid arthritis response criteria and patient-reported improvement in arthritis activity: is an American College of Rheumatology twenty percent response meaningful to patients?. Arthritis Rheumatol.

[30] Buch MH, Smolen JS, Betteridge N, Breedveld FC, Burmester G, Dorner T (2011). Updated consensus statement on the use of rituximab in patients with rheumatoid arthritis. Ann Rheum Dis.

[31] Listing J, Gerhold K, Zink A (2013). The risk of infections associated with rheumatoid arthritis, with its comorbidity and treatment. Rheumatology (Oxford).

[32] Global tuberculosis reports [Internet]. World Health Organization [cited 28 Feburary 2021]. Available from: https://www.who.int/teams/global-tuberculosis-programme/tb-reports.

[33] Rutherford AI, Patarata E, Subesinghe S, Hyrich KL, Galloway JB (2018). Opportunistic infections in rheumatoid arthritis patients exposed to biologic therapy: results from the British Society for Rheumatology Biologics Register for Rheumatoid Arthritis. Rheumatology (Oxford).

[34] Garcia-Montoya L, Villota-Eraso C, Yusof MYM, Vital EM, Emery P (2020). Lessons for rituximab therapy in patients with rheumatoid arthritis. Lancet Rheumatol.

