# Supplementary material for: Efficacy and safety of HLX01 in patients with moderate-to-severe rheumatoid arthritis despite methotrexate therapy: a phase 3 study
Source: Arthritis Res Ther. 2022 Jun 10;24:136. doi: 10.1186/s13075-022-02821-x (PMC9185960; doi:10.1186/s13075-022-02821-x)
Supplement: Supplementary file 1 — Additional file 1: Supplementary Data S1. Pre-medications, concomitant medications and rescue treatments. Supplementary Data S2. Inclusion and exclusion criteria. Supplementary Data S3. Adverse events of special interest. Supplementary Data S4. Sampling timepoints for assessments of pharmacokinetics, immunogenicity and pharmacodynamics. Supplementary Table S1. The HLX01-RA03 Investigators and study centres. Supplementary Table S2. Subgroup analyses of ACR20 response rate at week 24 in the intention-to-treat population. Supplementary Table S3. Adverse events of special interest. Supplementary Fig. S1. Proportion of patients with remission or low disease activity at weeks 36 and 48. Proportion of patients with remission (DAS28 ≤2.6) or low disease activity (DAS28 ≤3.2) at (a) week 36 and (b) week 48 in the intention-to-treat population. Error bars represent standard error. DAS28: disease activity score of 28 joints. Supplementary Fig. S2. Change from baseline in patient-reported outcomes. Adjusted mean change from baseline in (a) PtAAP-VAS, (b) HAQ-DI scores, (c) SF-36 PCS, and (d) SF-36 MCS in the intention-to-treat population. Error bars represent standard error. HAQ-DI: Health Assessment Questionnaire-Disability Index; PtAAP-VAS: Patient’s Assessment of Arthritis Pain-Visual Analogue Scale; SF-36 MCS: Short Form 36 Health Survey mental component summary; SF-36 PCS: Short Form 36 Health Survey physical component summary. Supplementary Fig. S3. Serum concentrations of HLX01 over time. Mean serum concentration of HLX01 over time in the pharmacokinetic set, plotted on (a) a linear scale or (b) a semi-log scale. Error bars represent standard deviation. Supplementary Fig. S4. Serum concentrations of HLX01 over time stratified by antidrug antibody status. Mean serum concentration of HLX01 over time stratified by antidrug antibody status in the HLX01 group ([a] linear scale; [b] semi-log scale) or in the placebo group ([c] linear scale; [d] semi-log scale) in the pharmacok [file 13075_2022_2821_MOESM1_ESM.docx]

SUPPLEMENTARY MATERIAL

# SUPPLEMENTARY DATA

**Supplementary Data S1** Pre-medications, concomitant medications and rescue treatments

*Pre-medications*

Pre-medications consisting of paracetamol, methylprednisolone or equivalent doses of other glucocorticoids, and diphenhydramine hydrochloride were given as instructed or as per local standard care to reduce infusion-related reactions.

*Concomitant medications*

Permitted concomitant medications included stable doses of oral glucocorticoids (≤10 mg/day), non-steroidal anti-inflammatory drugs (NSAIDs), and non-NSAIDs analgesics.

*Rescue treatments*

Patients who had <20% improvement in tender joint counts and swollen joint counts at weeks 16 and 20 could receive rescue treatment starting from week 20, including addition of new NSAIDs, oral glucocorticoids and/or analgesics, or increased doses of these concomitant medications.

**Supplementary Data S2** Inclusion and exclusion criteria

### Inclusion criteria

Subjects must have met all following inclusion criteria to be enrolled in the study:

1. Subjects who volunteer to participate in the study and sign a written consent form, and are willing and able to follow the study protocol (e.g., able to understand and complete the questionnaire, follow the visit plan, and use the drugs).
2. Age ≥18 and ≤75 years.
3. Subjects who are diagnosed with moderate-to-severe active rheumatoid arthritis (RA) (disease activity score of 28 joints [DAS28]-C-reactive protein [CRP] >3.2) at screening with a course of disease of ≥6 months and ≥6 swollen joints (based on a 66-joint count) and ≥6 tender joints (based on a 68-joint count); if a joint is swollen and tender, then this joint should be included in both the swollen joint counts and the tender joint counts (except for artificial joints).
4. Methotrexate (MTX) inadequate responders: subjects must be currently receiving MTX 10–25 mg/week for ≥12 weeks and have been on a stable dose for ≥4 weeks before treatment initiation (day 1); MTX needs to be given at a stable dose throughout the study, unless dose adjustments are made for safety reasons.
5. The accepted RA treatments must meet the following criteria:
   1. Subjects are willing to receive oral folic acid therapy (≥5 mg/week or at a dose determined by local medical practice) or equivalent medications (concomitant medications necessary for MTX therapy) during the entire study, and the dose of folic acid or equivalent medications should be stable for ≥4 weeks prior to the start of study treatment (day 1).
   2. If the subject has been previously treated with conventional disease-modifying anti-rheumatic drugs (DMARDs) other than MTX: leflunomide should be discontinued ≥8 weeks prior to the start of study treatment (day 1), but if the subject has received standard cholestyramine wash-out therapy (cholestyramine 8 g given orally, three times daily for 11 consecutive days), then leflunomide should be discontinued ≥4 weeks prior to the start of study treatment (day 1); other DMARDs should be discontinued ≥4 weeks prior to the start of study treatment (day 1); use of these drugs are not allowed throughout the study.
   3. If the subject is being treated with *Tripterygium wilfordii*, total glucosides of paeony, sinomenine, or other traditional Chinese medicines for RA, these drugs should be discontinued ≥2 weeks prior to the start of study treatment (day 1); use of these drugs are not allowed throughout the study.
   4. If the subject is receiving oral glucocorticoid treatment, the dose should not exceed prednisolone 10 mg/day (or equivalent dose of other glucocorticoids), and the dose should be stable for ≥4 weeks prior to the start of study treatment (day 1) and remain stable throughout the 26-week treatment period (except for those receiving rescue treatment); if already discontinued, oral glucocorticoids should be discontinued for ≥2 weeks prior to the start of study treatment (day 1).
   5. Glucocorticoid treatment administered intra-articularly or by injection is not allowed within 6 weeks prior to the start of study treatment (day 1) or during study treatment (until week 26), except for methylprednisolone 80 mg administered intravenously prior to study drug infusion (as this is part of the study process).
   6. Any NSAIDs or non-NSAID analgesics must be on a stable dose for ≥2 weeks prior to the start of study treatment (day 1) and remain on a stable dose throughout the 26-week treatment period (except for those receiving rescue treatment); if already discontinued, NSAIDs should be discontinued for ≥2 weeks prior to the start of study treatment (day 1).
   7. Technetium [^99^Tc] methylenediphosphonate (Yunke) should be discontinued for ≥12 weeks prior to the start of study treatment (day 1) and should not be used throughout the study.
6. Women of childbearing potential (subjects without childbearing potential refer to female subjects who have been menopausal for ≥2 years or have undergone total hysterectomy, bilateral tubal ligation or bilateral salpingectomy and/or bilateral ovariectomy, or who have congenital infertility) must agree to take effective contraceptives during the study and 12 months following trial completion or termination.
7. Men whose partners are women of childbearing potential are required to take reliable contraceptives during the study and 12 months following the last dose of the study drug, and male subjects should not donate sperm during this period.

### Exclusion criteria

Subjects meeting any of the following criteria will not be enrolled in the study:

1. Subjects receiving prior treatment with tumour necrosis factor (TNF)-α antagonists, other biologics for RA, or targeted synthetic DMARDs (e.g., JAK inhibitor tofacitinib).
2. Subjects of American College of Rheumatology (ACR) functional status Class IV or being bedridden/wheelchair-bound for a long term.
3. Subjects with primary or secondary immunodeficiency in previous or current medical history, including those with known history of human immunodeficiency virus (HIV) infection and those who have been tested positive for HIV.
4. Subjects with moderate-to-severe congestive heart failure (New York Heart Association Class III or IV).
5. Subjects with interstitial lung disease (except mild).
6. Subjects who have evidence (with a tuberculosis test at baseline) or history of active, occult, or latent tuberculosis.
7. Subjects allergic to murine proteins or other antibodies.
8. Subjects with a history of malignancy, including solid tumours, haematological malignancies, and carcinoma *in situ* (except subjects with basal or cutaneous squamous cell carcinoma, cervical dysplasia or *in situ* grade I cervical cancer resected and cured ≥12 months prior to the screening visit).
9. Subjects who have received a live vaccine/attenuated vaccine within 12 weeks prior to the screening visit, or will receive a live vaccine/attenuated vaccine during the 48-week study and the safety follow-up period.
10. Any disease or treatment (including biotherapy) that, at the discretion of the investigator, may bring unacceptable risk to the subject.
11. Pregnant or lactating female subjects, or subjects who will be pregnant or lactating during the study period or within 12 months after the last dose of the study drug.
12. Subjects previously or currently suffering from inflammatory joint diseases other than RA (e.g., gout, reactive arthritis, psoriatic arthritis, seronegative spondyloarthropathies, and Lyme disease), or other systemic autoimmune diseases (e.g., systemic lupus erythematosus, inflammatory bowel disease, pulmonary fibrosis, Felty’s syndrome, scleroderma, inflammatory myopathy, mixed connective tissue disease, and any overlap syndromes).
13. Subjects with significant concomitant diseases, such as, but not limited to, nervous system, cardiovascular, renal, hepatic, endocrine or gastrointestinal diseases that would preclude subject participation at the discretion of the investigator.
14. Subjects who are anti-hepatitis C virus antibody positive at screening.
15. Subjects who are anti-*Treponema pallidum* (TP) antibody positive at screening.
16. Subjects who are hepatitis B surface antigen (HBsAg) positive at screening; subjects negative for HBsAg yet positive for hepatitis B core antibody must be further tested for hepatitis B virus (HBV) DNA, and only HBV DNA-negative (or below the lower limit of detection at local laboratories) subjects can be enrolled.
17. Subjects with any active infections (except fungal infection of the nail bed), or any serious infections requiring hospitalization or intravenous anti-infective treatment within 4 weeks before the screening visit; or subjects receiving oral anti-infective treatment within 2 weeks before the screening visit.
18. Subjects with a history of deep space/tissue infections (e.g., fasciitis, abscess, and osteomyelitis) within 52 weeks prior to the screening visit.
19. Subjects with a history of serious or opportunistic infections within the last two years at the discretion of the investigator.
20. Subjects with a history of chronic infections (e.g., chronic pyelonephritis, bronchiectasis, and osteomyelitis).
21. Subjects with any congenital or acquired nervous system, vascular, or systemic diseases that may affect any of the efficacy evaluations in this study, especially joint pain and swelling (e.g., Parkinson’s disease, cerebral palsy, and diabetic neuropathy).
22. Subjects with a history of/currently with alcohol or drug abuse within 52 weeks prior to the screening visit (at the discretion of the investigator).
23. Subjects who have received anti-integrin αV antibody or cell depletion therapy within 3 months or 5 half-lives (whichever is longer) prior to the screening visit. Subjects who have received B-cell depletion therapy (e.g., CD20^+^, or CD19^+^, or CD38^+^ cell depletion) should be excluded from the study.
24. Subjects who are intolerant to glucocorticoid injections or have contraindications to glucocorticoids.
25. Subjects with any of the following abnormal results of laboratory tests at screening (no medical supportive treatments [e.g., white blood cell-increasing drugs, anaemia drugs except folic acid, hepatoprotective and enzyme-lowering drugs, blood transfusions, etc.] are allowed within 2 weeks prior to screening):
    1. Aspartate aminotransferase >2 × upper limit of normal (ULN).
    2. Alanine aminotransferase >2 × ULN.
    3. Haemoglobin <8.0 g/dl.
    4. Absolute neutrophil count <1.5 × 10^9^/l.
    5. Platelet count <75 × 10^9^/l.
    6. White blood cell count <3 × 10^9^/l.
    7. Serum creatinine >1.5 × ULN.
26. Subjects who have participated in any clinical studies (within 12 weeks or within 5 half-lives of the study drug, whichever is longer) before the screening visit, or subjects planning to participate in other clinical studies during the study period.

**Supplementary Data S3** Adverse events of special interest

1. Grade 3 or worse infusion-related reactions.
2. Grade 3 or worse skin and mucous membrane disorders.
3. Grade 3 or worse cardiac disorders and vascular disorders.
4. Grade 3 or worse infections.
5. Allergic reactions.
6. Hepatitis B infections.
7. Malignancies.
8. New-onset active tuberculosis.
9. Progressive multifocal leukoencephalopathy.

**Supplementary Data S4** Sampling timepoints for assessments of pharmacokinetics, immunogenicity and pharmacodynamics

*Pharmacokinetics*

Blood samples for pharmacokinetic assessments were collected on days 1, 15, 169 and 183 (pre- and post-dose), and at weeks 12, 36 and 48.

*Immunogenicity*

Blood samples for immunogenicity assessments were collected on days 1, 15, 169 and 183 (pre-dose), and at weeks 4, 8, 12, 28, 32, 36 and 48.

*Pharmacodynamics*

Rheumatoid factor (RF) was assessed at baseline, every 4 weeks from baseline to week 12 and from week 24 to week 36, and at week 48. CRP and erythrocyte sedimentation rate (ESR) were assessed at baseline and every 4 weeks until week 48. Anti-cyclic citrullinated peptide (anti-CCP) antibodies were assessed at baseline and week 24.

# SUPPLEMENTARY TABLES

**Supplementary Table S1** The HLX01-RA03 Investigators and study centres

| **Investigator name** | **Study centre** |
| --- | --- |
| Xiaofeng Zeng | Peking Union Medical College Hospital |
| Ju Liu | Jiujiang No. 1 People’s Hospital |
| Xiumei Liu | First Hospital of Shanxi Medical University |
| Lijun Wu | People’s Hospital of Xinjiang Uygur Autonomous Region |
| Yi Liu | West China Hospital of Sichuan University |
| Xiangping Liao | Chenzhou First People’s Hospital |
| Huaxiang Liu | Qilu Hospital of Shandong University |
| Jiankang Hu | Jiangxi Pingxiang People's Hospital |
| Xin Lu | China-Japan Friendship Hospital |
| Linjie Chen | The First Affiliated Hospital of Bengbu Medical College |
| Jian Xu | First Affiliated Hospital of Kunming Medical University |
| Zhenyu Jiang | The First Hospital of Jilin University |
| Fu-ai Lu | The First Affiliated Hospital of Baotou Medical College, Inner Mongolia University of Science and Technology |
| Huaxiang Wu | The Second Affiliated Hospital Zhejiang University School of Medicine |
| Lingyun Sun | Nanjing Drum Tower Hospital |
| Meimei Wang | Zhongda Hospital Southeast University |
| Xiaoxia Yu | Cangzhou Hospital of Integrated TCM-WM · Hebei |
| Pingting Yang | The First Hospital of China Medical University |
| Qinghua Zou | The Southwest Hospital of Army Medical University |
| Baijie Xu | Jieyang People's Hospital |
| Hua Zhang | Zaozhuang Municipal Hospital |
| Cibo Huang | Beijing Hospital |
| Liqi Bi | China-Japan Union Hospital of Jilin University |
| Xiaoxia Li | Xuanwu Hospital Capital Medical University |
| Jianzhao Cheng | Xiangtan Central Hospital |
| Hua Wei | Northern Jiangsu People's Hospital |
| Lan He | First Affiliated Hospital of Xi'an Jiaotong University |
| Hao Zhang | The Third Xiangya Hospital of Central South University |
| Hongsheng Sun | Shandong Provincial Hospital |
| Zongwen Shuai | First Affiliated Hospital of Anhui Medical University |
| Jianhong Zhao | Jining First People's Hospital |
| Yang Li | The Second Affiliated Hospital of Harbin Medical University |
| Rongbin Li | The First Hospital of Qiqihar |
| Fengju Li | Puyang Oilfield General Hospital |
| Xiaomei Li | Anhui Provincial Hospital |
| Zhuoli Zhang | Peking University First Hospital |
| Wufang Qi | Tianjin First Central Hospital |
| Hongwei Du | Jinhua Municipal Central Hospital |
| Jingchun Jin | Yanbian University Hospital |
| Jian Wu | The First Affiliated Hospital of Soochow University |

**Supplementary Table S2** Subgroup analyses of ACR20 response rate at week 24 in the intention-to-treat population

|  | **ACR20, *n**/*n*^†^ (%)** | | **OR (95% CI)** |
| --- | --- | --- | --- |
|  | **HLX01 (*n* = 183)** | **Placebo (*n* = 92)** |  |
| RF |  |  |  |
| Positive | 105/167 (62.9) | 32/86 (37.2) | 2.9 (1.7, 4.9) |
| Negative | 6/16 (37.5) | 1/6 (16.7) | 3.0 (0.3, 32.2) |
| ADA |  |  |  |
| Positive | 6/11 (54.5) | 3/10 (30.0) | 2.8 (0.5, 16.9) |
| Negative | 105/171 (61.4) | 30/81 (37.0) | 2.7 (1.6, 4.7) |

*n** = number of patients achieving ACR20 response; *n*^†^ = number of patients in each subgroup (RF positive or negative; ADA positive or negative) of HLX01 group or placebo group. ACR: American College of Rheumatology; ADA: antidrug antibody; CI: confidence interval; OR: odds ratio; RF: rheumatoid factor.

**Supplementary Table** **S3** Adverse events of special interest

|  | **Placebo-controlled part**  **Up to week 24 (SS1)** | | **Extension part**  **From week 24 to week 48 (SS2)** | |
| --- | --- | --- | --- | --- |
|  | **HLX01**  **(*n* = 182)** | **Placebo**  **(*n* = 92)** | **Continue HLX01**  **(*n* = 161)** | **Switch to HLX01 from placebo (*n* = 81)** |
| Any adverse events of special interest | 10 (5.5) | 2 (2.2) | 7 (4.3) | 5 (6.2) |
| Infections and infestations |  |  |  |  |
| Infective pneumonia | 2 (1.1) | 0 | 1 (0.6) | 1 (1.2)^a^ |
| Urinary tract infection | 0 | 1 (1.1) | 0 | 0 |
| Shingles | 1 (0.5) | 0 | 0 | 0 |
| Fungal rhinosinusitis | 1 (0.5) | 0 | 0 | 0 |
| Postherpetic neuralgia | 0 | 1 (1.1) | 0 | 0 |
| Active tuberculosis | 0 | 0 | 1 (0.6) | 2 (2.5) |
| Abscess of peritonsillar tissues | 0 | 0 | 0 | 1 (1.2) |
| Latent tuberculosis | 0 | 0 | 1 (0.6) | 0 |
| Viral hepatitis | 0 | 0 | 0 | 1 (1.2) |
| Skin fungal infection | 0 | 0 | 0 | 1 (1.2) |
| Immune system disorders |  |  |  |  |
| Immediate hypersensitivity reactions | 3 (1.6) | 0 | 0 | 0 |
| General disorders and administration site conditions |  |  |  |  |
| Non-cardiac chest pain | 1 (0.5) | 0 | 0 | 0 |
| Cardiac disorders |  |  |  |  |
| Acute myocardial infarction | 0 | 0 | 1 (0.6) | 0 |
| Premature beat | 0 | 0 | 1 (0.6) | 0 |
| Respiratory, thoracic and mediastinal disorders |  |  |  |  |
| Throat irritation | 1 (0.5) | 0 | 0 | 0 |
| Nasal discharge | 1 (0.5) | 0 | 0 | 0 |
| Nasal congestion | 1 (0.5) | 0 | 0 | 0 |
| Eye disorders |  |  |  |  |
| Watering eyes | 1 (0.5) | 0 | 0 | 0 |
| Gastrointestinal disorders |  |  |  |  |
| Retching | 1 (0.5) | 0 | 0 | 0 |
| Nausea | 1 (0.5) | 0 | 0 | 0 |
| Neoplasms benign, malignant and unspecified |  |  |  |  |
| Pulmonary sclerosing pneumocytoma | 1 (0.5) | 0 | 0 | 0 |
| Vascular disorders |  |  |  |  |
| Hypertension | 1 (0.5) | 0 | 1 (0.6) | 0 |
| Investigations |  |  |  |  |
| HBsAg positive | 0 | 0 | 1 (0.6) | 0 |

^a^ This case was reported as fungal pneumonia. Data are presented as number (%) of patients. HBsAg: hepatitis B surface antigen; SS1: safety set 1; SS2: safety set 2.

# SUPPLEMENTARY FIGURES

**Supplementary Fig. S1** Proportion of patients with remission or low disease activity at weeks 36 and 48


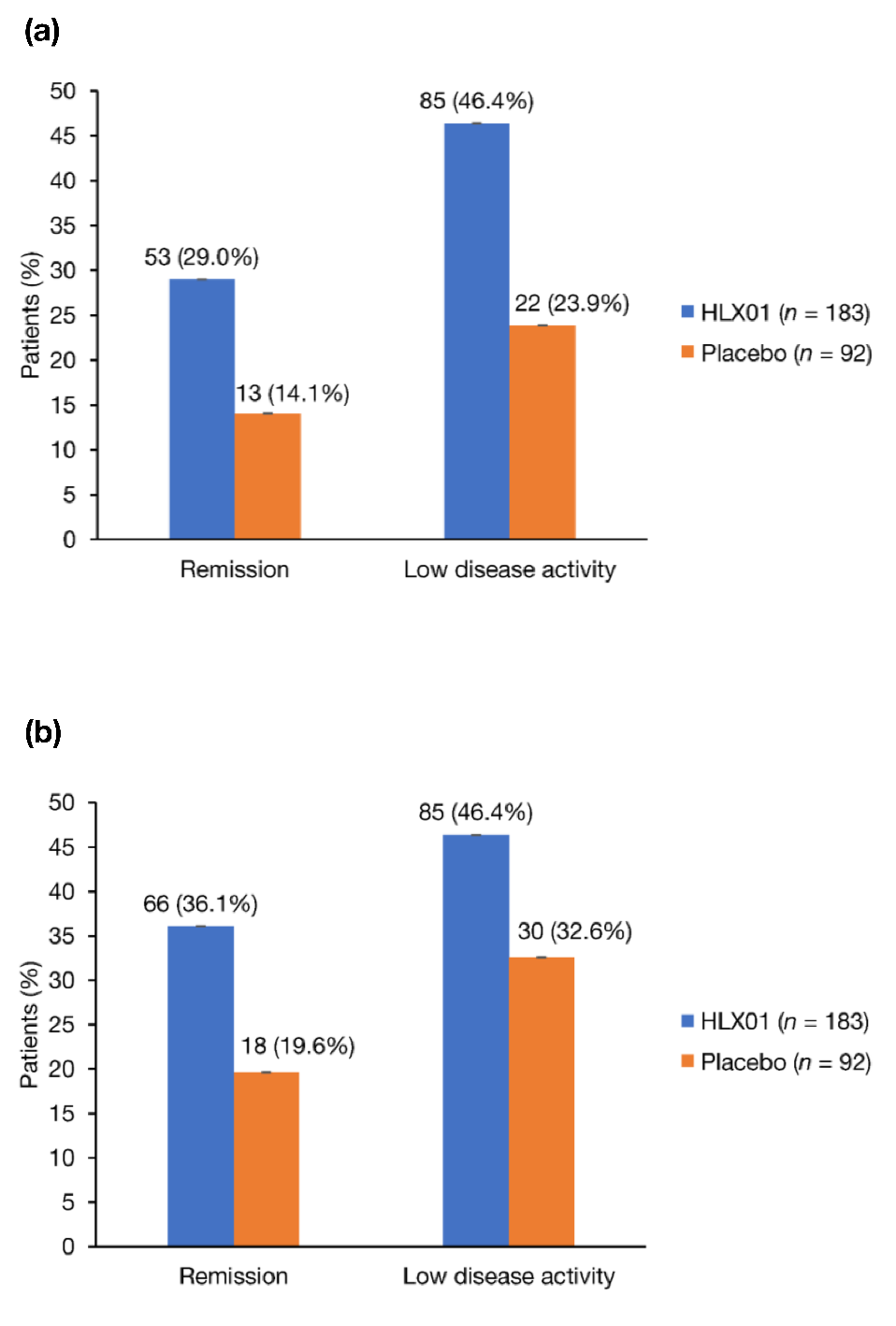


Proportion of patients with remission (DAS28 ≤2.6) or low disease activity (DAS28 ≤3.2) at (a) week 36 and (b) week 48 in the intention-to-treat population. Error bars represent standard error. DAS28: disease activity score of 28 joints.

**Supplementary Fig. S2** Change from baseline in patient-reported outcomes


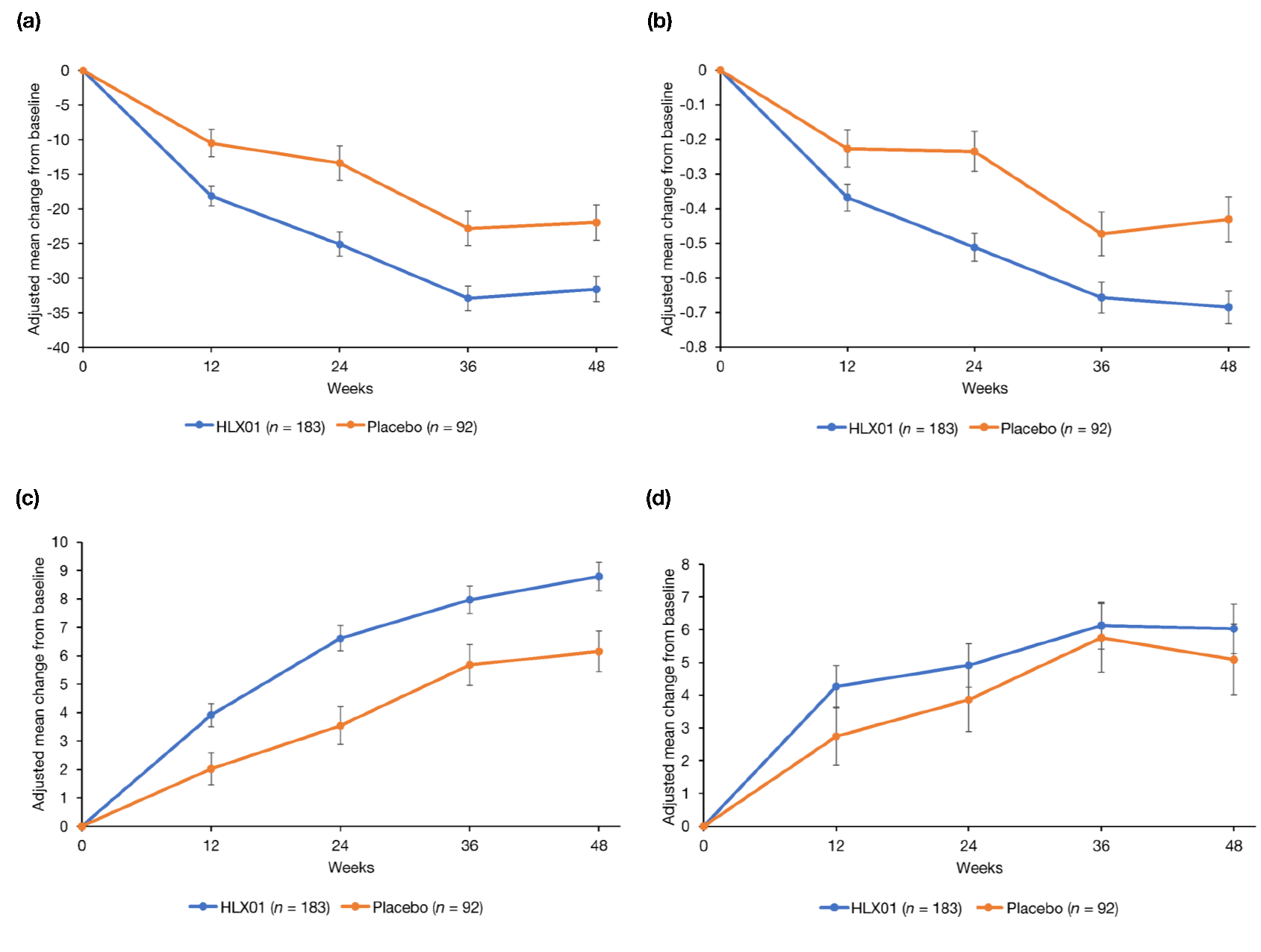


Adjusted mean change from baseline in (a) PtAAP-VAS, (b) HAQ-DI scores, (c) SF-36 PCS, and (d) SF-36 MCS in the intention-to-treat population. Error bars represent standard error. HAQ-DI: Health Assessment Questionnaire-Disability Index; PtAAP-VAS: Patient’s Assessment of Arthritis Pain-Visual Analogue Scale; SF-36 MCS: Short Form 36 Health Survey mental component summary; SF-36 PCS: Short Form 36 Health Survey physical component summary.

**Supplementary Fig. S3** Serum concentrations of HLX01 over time


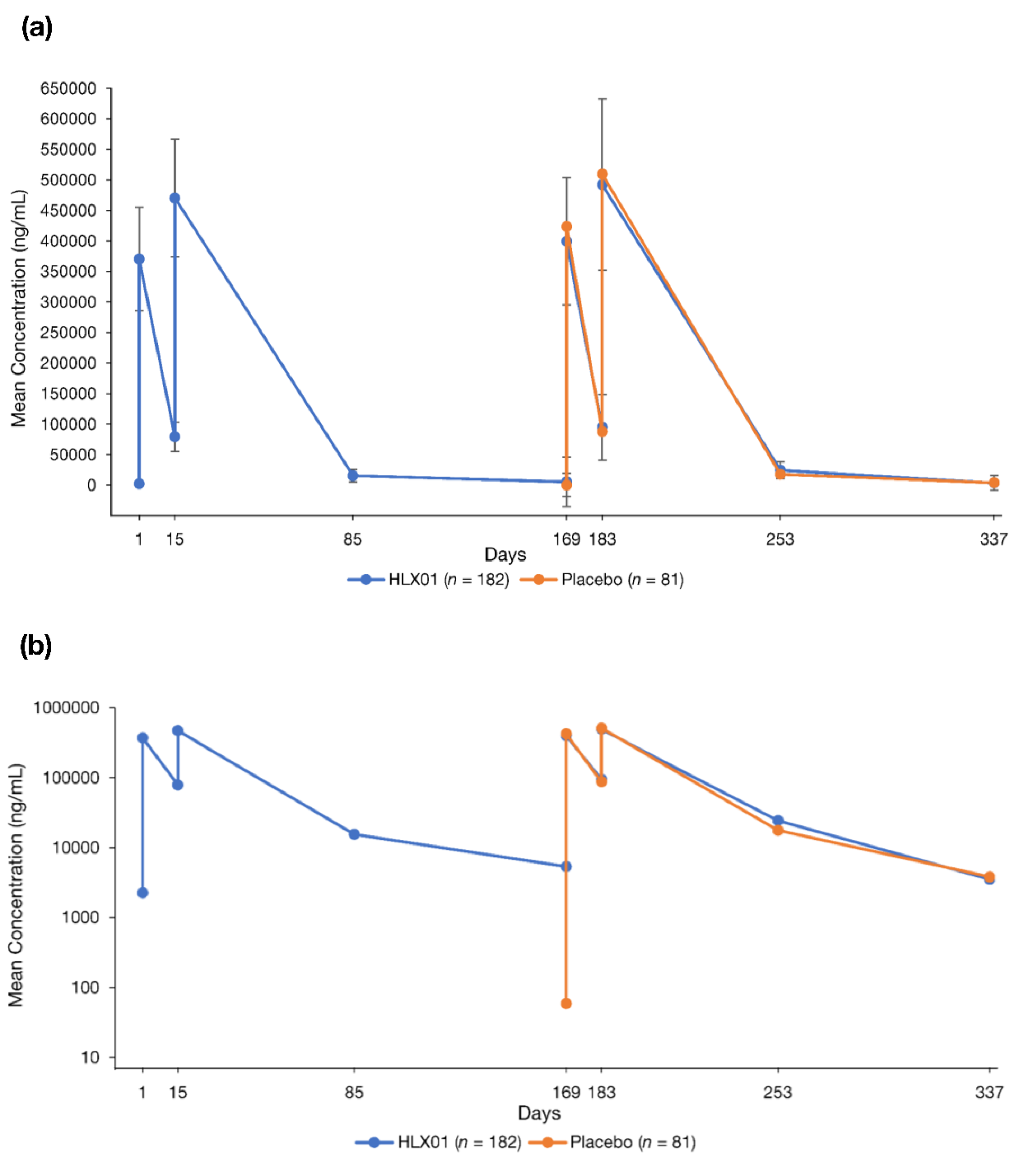


Mean serum concentration of HLX01 over time in the pharmacokinetic set, plotted on (a) a linear scale or (b) a semi-log scale. Error bars represent standard deviation.

**Supplementary Fig. S4** Serum concentrations of HLX01 over time stratified by antidrug antibody status


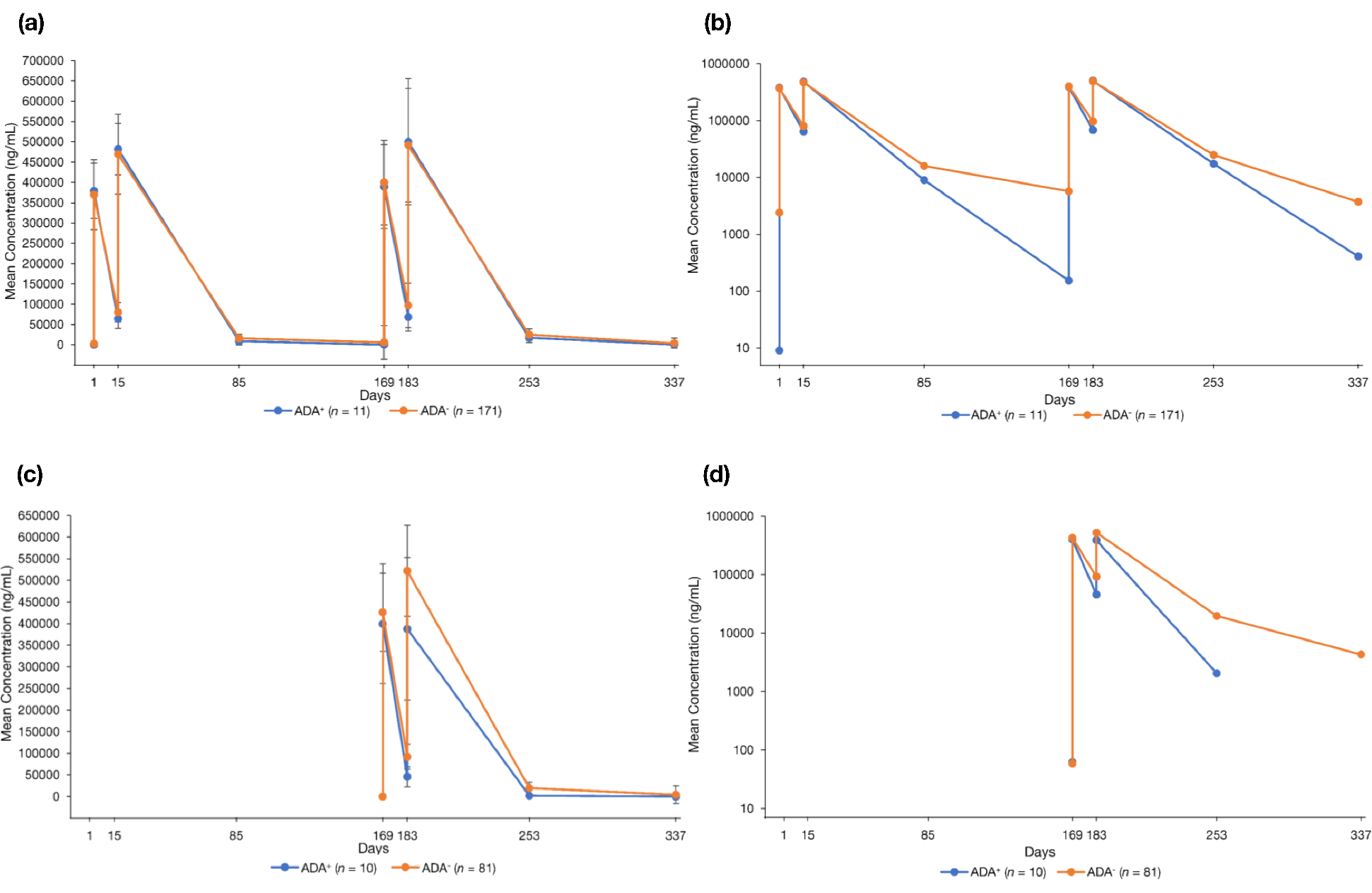


Mean serum concentration of HLX01 over time stratified by antidrug antibody status in the HLX01 group ([a] linear scale; [b] semi-log scale) or in the placebo group ([c] linear scale; [d] semi-log scale) in the pharmacokinetic set. Error bars represent standard deviation.

**Supplementary Fig. S5** Change from baseline in CRP and ESR over time


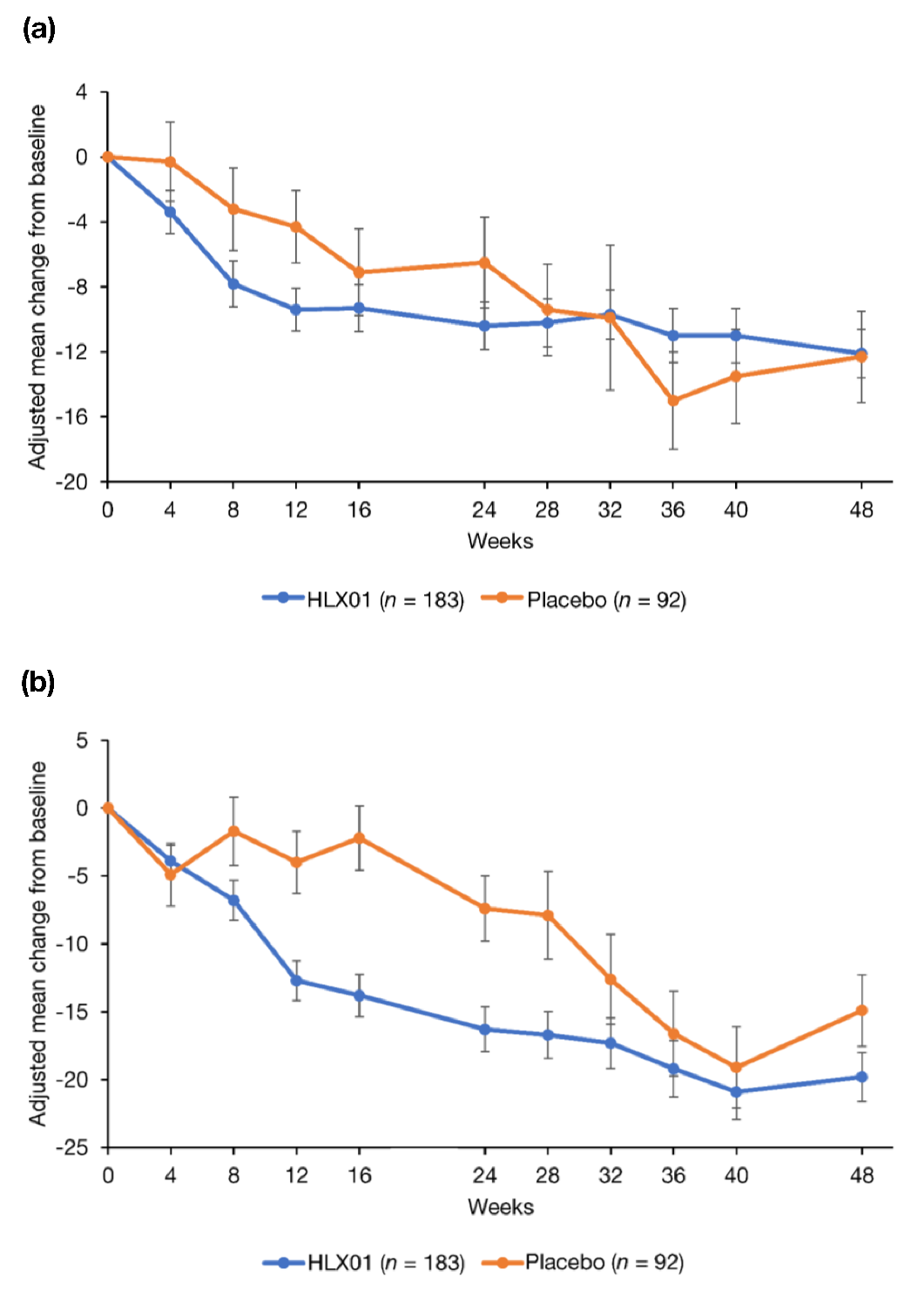


Adjusted mean change from baseline in (a) CRP and (b) ESR in the intention-to-treat population. Error bars represent standard error. CRP: C-reactive protein; ESR: erythrocyte sedimentation rate.
